# Sociodemographic Risk Factors of Diabetes and Hypertension Prevalence in Republic of Korea

**DOI:** 10.4061/2010/410794

**Published:** 2010-06-21

**Authors:** Hosik Min, JongWha Chang, Rajesh Balkrishnan

**Affiliations:** ^1^Center on the Family, University of Hawaii at Mānoa, Honolulu, HI 96822, USA; ^2^Department of Social and Administrative Science, College of Pharmacy, University of Michigan, Ann Arbor, MI 48104, USA; ^3^Center for Medication Use, Policy and Economics, University of Michigan, Ann Arbor, MI 48104, USA

## Abstract

This study examined the relationships between SES and diabetes and hypertension for Korean adults using the Korean National Health and Nutritional Examination Survey. To handle the four dummy dependent variables: Diabetes and Hypertension, Diabetes alone, Hypertension alone, and Diabetes or Hypertension, four different logistic models were conducted. The descriptive statistics showed a considerable amount of comorbidity between the combined dependent variable of diabetes and hypertension. To gauge more realistic measures of SES, education and income were combined together as four dummy categories. The SES factor indeed had significant impacts on diabetes and hypertension. Socioeconomically disadvantaged groups demonstrated to have increased likelihood of having these diseases. However, we could not find the strong compensating effect between education and income; the higher level of education but lower income variable was only significant in having both diseases, and the higher income but lower level of education variable was only significant in having hypertension alone and either one of the diseases. Only the highest SES one, the one with a higher level of education and a higher income, was significantly lowering the likelihood of having these diseases in all models. Therefore, public policy and intervention programs should focus on individuals matching these socioeconomic characteristics.

## 1. Introduction

Diabetes and hypertension are very common diseases in Republic of Korea as well as in other developed countries [1], on average, around 8.0% of Koreans reported that they had diabetes in 2007, and around 28% of Koreans reported that they had hypertension [2–5]. In addition, the prevalence for both diseases is increasing [5, 6].

Although diabetes and hypertension are not among the top leading causes of death, such as cancer and stroke, these two diseases draw attention from the public due to their increasing trends, while cancer and stroke are declining. Even diabetes has been ranked the 6th leading cause of death and is known as costly disease [7]. The prevalence of diabetes and hypertension increases with age [8]. Given the fast pace of population aging in Republic of Korea, one can assume that their prevalence will continue to rise [9].

In addition, these two diseases have been known for their high comorbidity [10–13]. It has been estimated that 20%–60% or even more of diabetic complications can be attributed to hypertension [11, 13]. Also, the prevalence of hypertension in diabetic individuals appears to be 1.5 to 3 times higher than in nondiabetic age-matched groups [11, 13]. Although diabetes is associated with considerably increased cardiovascular risk, the presence of hypertension in the diabetic individual markedly increases morbidity and mortality. People with both diabetes and hypertension have approximately twice the risk of cardiovascular disease as nondiabetic people with hypertension [12, 14].

Generally, both diseases are more common among males, the aged, and unmarried, and those who are less educated, and earn less are also at higher risk [10, 15–17]. It is not clear as to whether the place of residence is associated with diabetes and hypertension in combination. 

Despite the clinical importance of the coexistence of diabetes and hypertension as comorbid conditions, there are not many studies done on these two diseases taken together in Republic of Korea. Furthermore, although the relationship between socioeconomic status (SES) and health has been well documented, there is a lack of clear understanding about the combined impact of different SES measures, for example, education and income. For instance, what if we take education and income together? Although these two variables have often been analyzed independently, they are interrelated each other at an individual level in reality. Min had used these two variables as one composite variable and reported the supportive roles between education and income in Hawaii. A person with lower income but has higher education is still less likely to have both diseases; a person with lower education but has higher income is still less likely to have both diseases [18]. 

Therefore, the goal of this paper is to examine the relationship between the education-income composite SES variable with these two diseases. It is hoped that the findings of this study will contribute to the current body of knowledge by elucidating to the socioeconomic status that is associated with diabetes and hypertension, particularly among the Korean adult population and help policymakers, program planners, and other agencies to create more effective interventions.

## 2. Materials and Methods

The data used in this paper were obtained from the 2005 Korea National Health and Nutrition Examination Survey (KNHNES). The KNHNES is a representative sample survey based on household. The KNHNES survey instrument is modeled after the National Household Interview Survey (NHIS) conducted by the National Center for Health Statistics (NCHS), and all survey respondents are adult residents of Republic of Korea and supply information on all members of the household. The stratified sampling process was employed; a whole nation was divided 13 geographical regions following the census criteria; stratified again by 600 smaller administrative units; and then, 20–26 households were selected including the alternative households in case some households reject to participate in the survey. As a result, a total of 25,215 households' information was collected out of 25,487 sampled households; in other words, 98.9% of the sample was completed. The principle objective of the survey is to provide nationwide estimates of population parameters that describe (1) the current health status of the population; (2) the current physical status of health; (3) the nutrition status; (4) the distribution of the population by age, sex, and other fundamental demographic characteristics [19]. 

As described before, several sociodemographic factors, such as age, sex, marital status, education, and income have been known as risk factors [10, 15–17]. Special attention, however, is given to the SES, which is the focus of this study. The SES is defined as a measure of an individual or family's relative economic and social ranking [20], which is often measured by education, income, occupation, and so on [21–23]. Prior studies have reported their positive relationship on health; for example, the more educated, the better health status [5, 15–17, 21, 24]. But this study would create the composite variable of SES by combining education and income to gauge the complexity of SES. In other words, we do know both education and income positively affect on health, but we do not know how the relationship would turn out when we consider two variables at once. As Min found their study, would education and income show their supportive roles? May a person, who does not have a higher level of education but has adequate financial resources, have lower likelihood of having these two diseases? May a person, who lacks adequate financial resources but has a higher level of education, have lower likelihood of having these two diseases? 

In addition, there exist two possible statistical reasons to use these two variables as a composite variable. One is multicollinearity; more education level usually brings higher income, which implies a high correlation between two variables [25]. Second, income is measured at the household, not individual level. Thus, to avoid multicollinearity and gauge SES more accurately, the two variables were combined into one, which was done successfully in other study [18]. 

To do the analysis, two dependent variables, diabetes and hypertension, were combined first as a single variable, and then this new variable was constructed as four categories: diabetes alone, hypertension alone, both diabetes and hypertension, and either diabetes or hypertension. In other words, persons with diabetes were divided in two groups, those who had diabetes but not hypertension and those who had both diabetes and hypertension. This process was also followed for those with hypertension; then this new variable was automatically constructed as the four categories as described above. Both diabetes and hypertension were measured by the respondents' answers for their household members [19]: Questions were “Have you ever been diagnosed as having diabetes by doctors? And “Have you ever been diagnosed as having hypertension by doctors?” The four categories in the dependent variable were then created as four dummy variables, and four logistic regression models were conducted accordingly to handle the binary dependent variable [26]. 

There were seven independent variables was used to predict diabetes and hypertension, including age, sex, marital status, socioeconomic status (SES), and place of residence. All variables except age were coded in the following manners: female (0 = male, 1 = female), marital status (0 = unmarried, 1 = married), high school education-higher income, college education-lower income, college education-higher income (with high school education-lower income used as a reference group), and rural residence (urban area = 0, rural area = 1). As for SES, education and income variables were coded as a dummy variable: whether a person completed some college or more (yes = 1) and whether a person's household income per month is over 2,000,000 won, the median household income in these data, in Korean currency (around $1,800), the higher household income category (yes = 1). The two variables are combined in four categories and then recoded as four dummy variables, namely, high school education with lower household income (under 2,000,000 won), high school education with higher household income (over 2,000,000 won), college education (including some college) with lower household income, and college education with higher household income. Median household income has been used here to gauge the income that lies in the exact 50th percentile. By doing this, we can divide a population into two categories; earns more than that of the 50th percentile and earns less than that of the 50th percentile. Median household income compared to average household income is not affected much by the extreme categories like millionaires and billionaires [27]. 

## 3. Results

The descriptive analysis showed an adult prevalence of diseases under the four aforementioned categories. Only 2.9% of Republic of Korea reported that they had diabetes alone, 11.9% of them had hypertension alone, 3.0% of them had both diseases together, and 17.8% of them had either diabetes or hypertension. It is important to note that actual diabetes patients were 5.9% (=2.9% + 3.0%) and hypertension patients were 14.9% (=11.9% + 3.0%), because “both” means respondents had diabetes and hypertension at once. In other words, 51% of diabetes patients had both diseases (3.0/(2.9 + 3.0)* 100 = 51), and 20% of hypertension patients had both (3.0/(11.9 + 3.0)* 100 = 20). It implies that diabetes patients are more prone to have both diseases compared to hypertension patients. Thus, the extent of comorbidity between two diseases is more serious in diabetes, as Min found [18]. 

Table 1 presents the demographic characteristics of seven independent variables for the weighed 36,378,489 respondents in the KNHNES. It indicates that the average years of age for the Koreans is 43.2, half of them are females (50%), around two thirds of them are married (65%). On SES, only 31% of them have high school education with higher household income, 13% of them have college education with lower household income, and 26% of them have college education with higher household income. Around one out of five Koreans resides in rural area (18%). 

Table 2 presents the results of the four logistic regression models. The first column of Table 2 presents the analysis of the log odds of having diabetes and hypertension together. The first logit coefficient shown in the first column of Table 2 is 0.07 for age. This means that for every year's increase in age there is an increase of 0.07 in the log odds of having diabetes and hypertension. 

The estimated parameter effects are more straightforwardly interpreted when converted into odds ratios, which is done by exponentiation of the coefficients. Sometimes odds ratios in logistic regression model are referred to as “relative risk ratios”; that is, the relative risk, or the odds, of being in the dependent variable category of interest (i.e., dummy independent variable) and not being in the contrast or reference category of the dependent variable [26]. The odds ratio for age is *e* ^0.07^ = 1.07, which means that the odds of a person having both diabetes and hypertension must be multiplied by 1.07 as being an old, which means that the odds increase with age. We can determine the percentage amount of change in the odds by subtracting 1 from the odds ratio and multiplying the difference by 100. Thus, we arrive at (1.07−1)* 100 = 7, indicating that the odds of having both diseases increases 7% more with additional increase of age. This positive pattern can be found in the other three models.

The next is for the SES variables, which is our focus variable. Among all the SES variables, only the highest SES category, college education with higher income, provided consistent advantages over these two diseases; 57% less for both diseases, 38% less for diabetes alone, 18% less for hypertension alone, 24% less for either one, respectively. College education and lower income is only significant at hypertension alone and either one, 27% less and 22% less, respectively. Lower education and higher income is only significant at both diseases, 19% less likely to have both diabetes and hypertension. Conclusively, higher education seemed to work well with both lower and higher income only in hypertension alone and either one, not other models. Meanwhile, higher income seemed to work well only in both diabetes and hypertension, but not for the other three models. For diabetes alone model, only the higher education and higher income category is significant. Thus, education seemed the largest impact of the highest SES on having both diseases which means that this category is the most vulnerable on SES. These results imply that education and income only show limited reciprocal supportive roles.

Females are less likely to have either or both of these diseases. For instance, the odds ratio for diabetes alone is 0.60. This means that female Koreans are 40% less likely to have diabetes alone as compared to their male counterparts. This negative pattern for female also can be found in other models except hypertension alone. 

Married adults, as compared to unmarried ones, show a higher likelihood of having diabetes alone (48% more), hypertension alone (25% more), and diabetes or hypertension (28% more), but not in both diseases. It appears to be that being unmarried confers an advantage for these two diseases, which is against the common expectation. 

Interestingly, rural dwellers show an advantage over these diseases except diabetes alone; for instance, people who lived in rural area are 24% less likely to have diabetes and hypertension; 13% less for hypertension alone and either diabetes or hypertension, respectively. 

## 4. Discussion

The goal of this paper was to examine the relationships between SES and diabetes and hypertension in Republic of Korea. As expected, the combined dependent variable of diabetes and hypertension shows a considerable amount of comorbidity, particularly in diabetes; more than 50% of people diagnosed as having diabetes also have hypertension, on the contrary, only 20% of people with hypertension also have diabetes. The comorbidity is a serious concern because people with both diseases have mortality rates that are two or three times higher than patients with diabetes alone [11, 13]. Another concern is population aging in Republic of Korea: Due to Republic of Korea's very low fertility rate [28], longer life expectancy and low mortality, the pace of population aging is one of the fastest in the world [29, 30]. Thus, considering this population aging in Republic of Korea with the fact of the growing patterns of diabetes and hypertension, one can expect that this problem will be more pervasive. 

As described before, no direct study has been made on this diabetes and hypertension comorbidity taken together in Republic of Korea. The results showed that the socioeconomic status indeed played an important role in these two diseases in Republic of Korea as in Min's study, but with limitation. For instance, a person with a higher level of education and a higher income was less likely to have these diseases in all four models. In addition, a person who had a higher level of education but with a lower income was less likely to have hypertension alone and either one, not both diseases and diabetes alone. In addition, a person who had a higher income but had a lower level of education was less likely to have both diseases alone, but this relationship was not significant with other models. Education and income in Republic of Korea did compensate each other to avoid having these two diseases, but not as strong as in Hawaii population. In short, people who are socioeconomically disadvantaged are the most vulnerable in having both diseases; but the compensational effect between education and income to avoid having these diseases cannot be seen in all categories. In other words, the compensational effect is weaker in Republic of Korea compared to that of Hawaii. Also, we may conclude that a person with a higher education and a higher income only can be benefited in Republic of Korea.

Most of other covariates in the models were also were significant. The associations between dependent and independent variables also showed as expected ways except marital status and rural residence. A person is more likely to have these diseases with an increase of age; females are less likely to have these diseases. Married person, however, showed a positive relationship with these diseases. To see if a married person is really vulnerable on these two diseases, this study conducted an additional analysis by creating an interaction variable; sex and marital status. The results showed that the sex and marital status interaction variable was only significant in the diabetes or hypertension model, not others. One cannot conclude that marital status is a real contributing factor on having these diseases. Otherwise, considering the close relationship between age and marital status; the age, the more likely to be married, marital status would be more closely related to the age. Because marital status variable is used as a controlling one, however, the results of further analysis on these variables were not shown in this report. As described before, we do not have a clear understanding of rural/urban residence. This study, however, showed a positive relationship between rural residence and these two diseases only except diabetes alone. It may also relate to the lifestyles and diet of rural Korean dwellers: more physical activities, healthier natural environment, less fast food, and more vegetables. This also requires further study.

Accordingly, the policy and intervention programs for the reduction of diabetes and hypertension in Republic of Korea should include SES. As seen in Table 2, a person who had both a higher level of education and a higher income is the most benefited; the compensating effect between education and income is not as strong as in other countrios. People who are soecioeconomically disadvantaged and who lack financial resources and/or education, which is expected to convert to have better or more knowledge on health, need more attention. In addition, given the fact that these diseases increase with age [9] and the fast pace of population aging in Republic of Korea [29, 30], age, in particular the elderly, should be taken into account as one of the important contributing factors on diabetes and hypertension. Therefore, public policy and intervention programs should focus on individuals matching these sociodemographic characteristics.

## Figures and Tables

**Table 1:** Descriptive statistics (*n* = 36,378,489; weighted).

Variable	Mean	S.E	95% C.I.	
Age	43.20	0.103	42.99	43.40
Female	0.50	0.003	0.49	0.51
Married	0.65	0.003	0.64	0.65
*SES*				
High school education-higher income	0.31	0.003	0.30	0.32
College education-lower income	0.13	0.002	0.12	0.13
College education-higher income	0.27	0.003	0.26	0.27
Rural Residence	0.18	0.001	0.18	0.19

**Table 2:** The Results of Logistic Regression Models.

	Diabetes and hypertension		Diabetes alone		Hypertension alone		Diabetes or hypertension	
Variable	OR (Coef.)	95% CI	OR (Coef.)	95% CI	OR (Coef.)	95% CI	OR (Coef.)	95% CI
Age	1.07 (0.07)	[1.06, 1.08]**	1.04 (0.04)	[1.03, 1.05]**	1.07 (0.07)	[1.06, 1.07]**	1.07 (0.07)	[1.06, 1.07]**
Female	0.83 (−0.18)	[.70, .99]**	0.60 (−0.52)	[.50, .70]**	1.00 (0.00)	[.91, 1.09]	0.87 (−0.14)	[.80, .95]**
Married	0.93 (−0.07)	[.78, 1.11]	1.48 (0.39)	[1.02, 1.81]**	1.25 (0.22)	[1.12, 1.39]**	1.28 (0.25)	[1.16, 1.42]**
*SES*								
High school education & higher income	0.81 (−0.21)	[.66, .98]**	0.98 (−0.02)	[.81, 1.18]	0.98 (−0.01)	[.88, 1.09]	0.98 (−0.02)	[.89, 1.07]
College education & lower income	1.01 (0.01)	[.70, 1.45]	0.90 (−0.11)	[.63, 1.28]	0.73 (−0.31)	[.58, .89]**	0.78 (−0.25)	[.64, .93]**
College education & higher income	0.43 (−0.85)	[.29, .61]**	0.62 (−0.48)	[.46, .82]**	0.82 (−0.19)	[.71, .96]**	0.76 (−0.27)	[.67, .88]**
Rrual residence	0.74 (−0.30)	[.61, .90]**	0.89 (−0.12)	[.73, 1.07]	0.87 (−0.14)	[.78, .96]**	0.87 (−0.14)	[.78, .96]**
constant	−6.89		−5.68		−5.64		−5.28	

Number of stratum	36		Population in size		36,378,489			
Number of PSU	600		Design (df)		25,158			
Number of observation	25,194							

**P < .05.

OR: Odds Ratio.

Coef.: Coefficient.
